# Inaugural Dissertation on the Influence of Climate on the Health and Mortality of the Different Regions of the Globe

**Published:** 1838-07

**Authors:** 


### ART. XI.

*Inaugural Dissertation on the Influence of Climate on the Health and Mortality of the different Regions of the Globe.* By A. S. THOMSON, Candidate for the Degree of Doctor in Medicine.—*Edinburgh*, 1837. 8vo. pp. 102.

THIS essay is a prize thesis in English. It appears that the writers of the greater part of the theses at the late graduations in Edinburgh have substituted the English for the Latin language: this, as we remarked on a former occasion, is a result to be expected from the regulation which renders the choice of the language optional with the candidate. We repeat, that we are not without fears that the new method may have the effect of lessening the amount of classical knowledge among the candidates for the doctorate. Still we are ready to admit that the old system was attended with very serious inconveniences; and, if the great superiority of the theses presented on the new plan is to be regarded as a proof of its superiority, it is impossible to resist the argument. To judge from such of the theses as have been printed, the improvement in the subjects, the matter, and the style has been very great. If we demurred to the propriety of having the thesis in English, we had no doubt of the necessity of having the principal part of the examinations in the vernacular tongue. Making Latin the language of the examination tended very much to render the whole a mere matter of form; and, as it was an easy step from procuring a translation to purchasing a thesis, we fear this step was often taken. These circumstances contributed not a little to the partial discredit into which inaugural dissertations have fallen as a test of mental capacity; yet this discredit is, in our opinion, unmerited. An oral or written examination may show the candidate's range of acquirements, the tenacity of his memory, and the readiness with which he can call up his knowledge, and bring it to bear upon any given question; but it does not fully display the original powers of his mind. An essay, where he has access to books, and time for observation, experiment, meditation, calculation, on the other hand, effectually answers this purpose. A practical examinator can form an accurate opinion, from the thesis, of a man's industry and acuteness in collecting facts, and arranging them in their natural relations: nor would it be so difficult as some imagine to identify the thesis as the candidate's own production, or to discern the common-place essays of venal writers, if proper precautions were taken. In directing attention to a special point of enquiry, the thesis also often lays the train of important subsequent results: several of our best works are known to have sprung from this source. Perhaps the candidate should not be put to the expense of printing against his wish; although the publication of a good thesis would be the best justification imaginable of the examining body, and of the title it conferred. The thesis, in our opinion, cannot be safely superseded: and we hope its presentation, at least, if not publication, will be among the regulations adopted by the senate of the new London university.

Dr. Thomson has dedicated his thesis to Henry Marshall, Esq., "who (he gratefully adds) suggested the subject, and from whose statistical researches a great part of the materials have been obtained." This, while it does not detract from Dr. Thomson's merit, raises the value of the work; as the character of Mr. Marshall's valuable labours in the medical statistics of the army is well established.

The author has not examined climate under the same point of view as Sir James Clark, in his classical work on that subject; he has treated its elements less as therapeutic agents than as causes of sickness and death, with reference rather to the prevention of diseases than their causes: but, as regard should always be had to the influence climate has in producing particular diseases, and in increasing or diminishing the mortality of natives and foreigners, no one can undertake to deal with climate, or to apply it in practice, not well versed in these particulars. Few will deny the necessity of this preliminary information, when they see patients sent in search of health to Jamaica, and other places notoriously unhealthy.

After a rapid sketch of the temperature, rain, and winds of the torrid zone, and of the various races of inhabitants, Dr. Thomson proceeds to examine the ratio of mortality among the indigenous population. It has been often stated that the mortality is greater among the inhabitants of the torrid zone than in temperate climates; but no positive observations have been produced to prove that the mean duration of life is really shorter between the tropics than elsewhere. The facts on which the statement set forth by M. Moreau de Jonnès rests have never been published; and, under these circumstances, do not deserve to be cited. The following observations appear to show that the mortality of the black troops on the west coast of Africa is above the average, but below the mortality of the black troops in the West Indies.

| Period of observation. | Mean number living. | Annual deaths per cent. |
|---|---|---|
| 14 years. | 469. | 3.6. |

In the island of Bourbon, Mr. Thomas states that the annual mortality of the slave population was 3 per cent.; among the 5069 free blacks, 1.6 per cent. The latter could not have included children. The annual rate of mortality in New Spain (tropical America) is 3.3 per cent. (*Humboldt.*) Lieutenant Tulloch has constructed an interesting table of the mortality among the slave population of the West-India islands, from which it appears that 2.8 per cent. die annually in twelve colonies taken together; and that the mortality in Tobago was 4.15 per cent., and in Antigua 2.4 per cent. The observations generally extended over fourteen years (1817-30), and embraced 249,037 deaths, 8,954,513 years of life. The mortality of the African troops appears to be higher than the mortality of the African slaves. In the nineteen years, 1810-28, 2,938 deaths took place out of 51,734 living one year: the mortality was 5.6 per cent., although the rules of the service protect the soldier from harsh treatment. Lieutenant Tulloch concludes, therefore, that the mortality and decrease of the slave-population in the West-Indies is due rather to the climate than to the miseries of slavery. It should be recollected that the mortality of soldiers is always higher than the mortality of the general population at the same age.

Of the black troops in the West Indies, (1817-31,) 30 per 1000 died annually in Jamaica; and, of these, 8 from fever, 10 from diseases of the lungs, 0.5 from diseases of the liver, 2.5 from diseases of the bowels. In the Windward and Leeward Islands, 40 per 1000 died annually; 4.4 from fever, 16.6 from diseases of the lungs, 1 from disease of the liver, 7.3 from diseases of the bowels. The negroes are, therefore, not exempt from fever, but it is much less fatal to them than to Europeans. Pectoral affections are by far the most destructive disorders among the African population in the West Indies; and this is to a considerable extent induced by the climate, if much confidence can be placed in an extravagant statement advanced by Dr. Winterbottom. Speaking of consumption among the native Africans in the vicinity of Sierra Leone, he says, "As an idiopathic disease, I do not recollect to have seen a single case!" However this may be, tubercular consumption is exceedingly fatal among the negro troops, not only of the West Indies, but of Ceylon and the Mauritius. (pp. 22-3.) Colonel Sykes stated, at the British Association, that the mortality in the Deccan (population, 3,000,000,) was 2.7 per cent. annually. In 1827-30, the annual deaths among 74,850 native troops amounted to 1010, or to 1.35 per cent. In the presidency of Bengal, the native troops, in 1833, amounted to 90,075, the deaths to 955; to 1.06 per cent.: in the presidency, the mortality was 5.8 per cent.; in Cawnpore, 0.7 per cent. In 1827-30, the deaths in the native troops of the Madras presidency amounted to 4,041; the deaths per cent. from fevers to 0.25, pectoral complaints 0.06, diseases of the liver 0.01, diseases of the bowels 0.11, cholera morbus 0.33; all diseases 1.35. The mortality from pectoral diseases is low: among the troops in Scotland it is 0.46 per cent., nearly eight times higher than among the native troops of

India. The proportion *constantly sick* is a very important point of enquiry: the only light thrown on this is derived from returns of the sick among the native troops.

*Sickness among the Native Troops of Bengal and Agra,* 1833.*

| Total strength. | Mean number constantly sick. | Sick per cent. | Deaths per cent. |
|---|---|---|---|
| 90,075 | 4,238 | 4.7 | 1.06. |

Dr. Thomson then investigates the number of cases of sickness and the influence of the seasons, which concludes the first chapter on the climate of the Torrid Zone.

The second chapter is devoted to the *Temperate Zones,* where the several branches are examined in the same order, but at greater length, as the materials are more abundant. The third chapter treats of *the Influence of a Temperate Climate on the indigenous Inhabitants of the Torrid Zone;* the fourth of the *Influence of an Intertropical Climate on the indigenous Inhabitants of the Temperate Zones.* Our limits do not allow us to make any further analysis of Dr. Thomson's work. The following facts, however, our readers will do well to compare with others previously cited.

*British Troops in Bengal and Agra,* 1833.†

| Strength. | Died. | Mean Sick. | Died per Cent. | Sick per cent. |
|---|---|---|---|---|
| 11,999 | 632 | 1,551 | 5.3 | 12.9. |

According to Dr. Burke, (1826-32,) the deaths, out of 60,904 living one year, amounted to 3,486, or 5.7 per cent.; namely, 1.54 by fevers, 0.25 by pectoral diseases, 0.42 by diseases of the liver, 1.84 by diseases of the bowels, 1.15 by cholera morbus. In the West Indies, (1817-34,) diseases prevailed among the British troops in very different proportions. In the *Windward and Leeward Islands,* the annual mortality was 8.0 per cent.; 3.86 from fevers, 1.05 from diseases of the lungs, 0.19 from diseases of the liver, 1.9 from cholera morbus. In *Jamaica,* the mortality was 13.1 per cent.; viz. 11.26 from fevers! 0.74 from diseases of the lungs, 0.8 from diseases of the liver, 0.45 from diseases of the bowels, 0.1 from cholera.

Dr. Thomson has not succeeded in showing the influence of a change northward on the inhabitants of tropical climates; a defect we shall endeavour to supply from a very interesting series of observations, published by Sir James Macgregor, in his Medical Sketches (1804.)

When the French were in possession of Egypt, an English army was assembled in the Mediterranean, to drive them from that country; and at the same time an Anglo-Indian army moved from the shores of the English possessions in the East. This army was 6,800 strong. The regiments disembarked at Cosseir Bay, in 1801, and arrived only in time to see the French capitulate, without having any share in the campaign. They remained nearly one year and a quarter in Egypt. There were 3,759 British troops; 3,042 Sepoys (Hindoos): the departments, 419, and public and private followers, 666, we exclude at present from calculation.

As the regiments landed in different months, it was necessary to reduce

* Report of Mr. Hutchinson on the Native Jails. † Ib.

the time to one year; which was done in the following manner: The 10th English regiment landed "28th November, 1800, and left Egypt 1st May, 1802:" it therefore remained in Egypt one year and five months, = 1.42 years. The strength, 984, multiplied by 1.42, would give 1,397 years of life: in other words, 984 men in Egypt one year and five months may be considered equivalent to 1,397 living there one year. During their residence in Egypt, 98 died, and 49 were invalided; half of these (73) should be deducted from 984 as a correction; but, as the deaths and invaliding occurred in greater proportion towards the latter end of the period, we have subtracted them from the years of life, 1,397, thus reduced to 1,324. The ten different English corps and the nine Indian corps were thus treated separately, and the following were the results:

| | BRITISH. | HINDOOS. | ANN. PROPORTIONS. | |
|---|---|---|---|---|
| | | | British. | Hindoos. |
| Strength on landing | 3759 | 3042 | | |
| Years of life | 4355 | 3551 | 100 | 100 |
| Invalided | 117 | 41 | 2.7 | 1.2 |
| Total deaths | 315 | 326 | 7.2 | 9.2 |
| Deaths by Plague | 38 | 112 | .9 | 3.1 |
| Fever | 18 | 85 | .4 | 2.4 |
| Liver Complaints | 64 | 10 | 1.5 | 0.3 |
| Dysentery | 148 | 42 | 3.4 | 1.2 |
| Diseases of Lungs | 4 | 4 | .1 | .1 |
| Stroke of the Sun | 2 | 0 | .05 | 0 |
| Casualties and other Diseases | 41 | 73 | .9 | 2.1 |

It will be recollected that the mortality of the Hindoo troops in India is 1.35 per cent.; and that the mortality of the British troops in India is about 5.5 per cent. In Egypt, "on the bleak shores of the Mediterranean," (Macgregor,) where the thermometer raged in December from 49° to 70°, the mortality of the Indians was 9.2 per cent. two per cent. higher than the mortality of the British engaged in active duty. The number invalided from the British troops was higher than the number invalided from the Hindoos; the casualties among the latter were more numerous; and two Bombay regiments of Sepoys were particularly exposed to the causes of fever and plague. It may, therefore, be admitted that, *cæteris paribus*, the mortality would be nearly the same in the troops of both races. But what a remarkable and characteristic difference in the disease by which they were attacked and decimated! The Indians fell chiefly by plague and fever: the Europeans by dysentery and liver diseases. One of the English regiments came from the Cape of Good Hope; the others from Calcutta, Bombay, &c.; but the difference of mortality on diseases in these cases was not considerable. Among the Indians, the mortality bore principally on the 1st and 7th Bombay regiments; the 7th, a corps of 708 men, lost 69 by plague, 42 by fever; in all, 160 by death, 10 by invaliding, in 1¼ year. This regiment was harshly used. On the voyage, it had suffered severely from fever; the transports having been probably crowded. While the rest of the army proceeded to salubrious quarters at Alexandria, the 7th Bombay regiment was left at Rosetta, where the plague raged violently, till January 8th: it was then marched to Aboukir Bay, to remain isolated with a pest establishment. The Indians gave themselves up to despair; and, when conveyed to the Pest-House, would take neither food nor physic. The mortality in the departments, consisting of 419 natives, was low: not more than 25 deaths (10 of plague) out of 578 years of life, equal to an annual mortality of 4.3 per cent. The deaths (forty) among the public, and private followers (666), were equally few; showing that half the mortality was due to the peculiar exposure and hardships of the common soldiers on active duty.

The march of the Anglo-Indian army over the desert of Thebes, and the descent of the Nile, excited a great sensation at the time; and we are not aware that any other statistical observations exist showing the influence of climate on the Hindoo race. To make this notice complete, it may therefore be well to recall a few of the circumstances recorded by Sir J. Macgregor, in his Journal of the Expedition.

To carry the men from India, large, lofty, and roomy ships were fitted up, and the greatest care was taken to embark *only such as were in perfect health.* Excellent provisions and wine for the sick, were provided. The army landed at Cosseir Bay, and penetrated Egypt through the desert of Thebes. In *June*, 1801, there was little sickness; in *July*, the army at Ghenne suffered more; on *August* 12th, great part of the army, after a navigation of the Nile of nearly 400 miles, arrived at Ghiza. The troops were uncommonly healthy on landing: in a week, most of the corps sent eight to ten per cent. of their strength to hospital. In three weeks, there were 1000 sick, chiefly of fever. The cases of ophthalmia were also numerous. Ghiza was found to be swampy. At the end of August, the army embarked for Rosetta: sick 1200. Rosetta is surrounded on two sides by swamps. The streets are narrow, dirty lanes, with high houses, overhanging each other, and the passage frequently interrupted by houses in ruins. Here, during *September*, the sickness gained ground rapidly; particularly ophthalmia, dysentery, and hepatitis. The first cases of plague appeared; and the cases of ophthalmia amounted to 600. In *October*, the army removed 1-5 miles distant from Rosetta; *November* 1st, sick 1350, or more than 25 per cent.; 170 cases of intermittent fever occurred. Sick in the weekly return, November 20-26, Europeans, 632, Sepoys, 380, = 1012. *December*, nearly all the army marched to Alexandria. Several cases of plague and continued fever occurred; and, towards the end of the month, many cases of catarrh, pneumonia, rheumatism, and ophthalmia. December 3d, 312 cases of ophthalmia; December 31st, 98. January, 1802, the mortality increased; the number of sick was smaller: 92 cases of plague occurred. *February*, 1802, fever, hepatitis, dysentery decreased; 21 cases of plague occurred. February 1st, sick 705. February 28th, 312. *March*, small-pox broke out among the sepoys: total sick of the army, 300. The Indian army was now ordered to return. It marched by night over the desert of Suez, encamping at sunrise. It suffered several times from the dry suffocating wind of the desert; which, at Moses's Wells, caused the thermometer to rise from 60 to 98 degrees: ophthalmia became more frequent. On June 2d, 1802, the embarkation commenced. Most of the corps were in a healthy state, except the 7th regiment (Indian), which was ordered to remain two months. No cases of plague had occurred after the army reached India, so late as November, 1802.

Besides the diseases in the table, the Indian army suffered severely from ophthalmia; it sent home fifty invalids from blindness. Few officers were attacked. In the 88th, (strength 466,) where forty men did not escape, only two out of thirty officers were attacked: one lost an eye. The Guinea-worm was little seen in Egypt: on the voyage 199 cases occurred among 360 men of the 88th.

The deaths in the French army, from May 30th, 1798, to September 30th, 1800, amounted to 8542;* 3561 happening in battle, 852 as the consequences of wounds; 281 died of accidents; 1429 of plague; 2419 of ordinary diseases. From other facts, which we cannot discuss here, it appears that the strength of the French army was 25,729. The annual mortality, therefore, was 14.2 per cent.: 5.9 per cent. were killed in battle; 1.4 died of wounds; 0.5 of accidents; 4.0 of common diseases; 2.4 of plague, = 14.2. Exclusive of the deaths from fighting, the annual rate of mortality was 6.9 per cent.: very nearly the mortality observed in the British troops. The annual mortality of the French army at home, including officers, is 1.94† per cent.; it was, therefore, increased more than sevenfold by the Egyptian campaigns, the climate, and the epidemic plague.

These facts authorize us to conclude that the mortality of inhabitants of the torrid and of the temperate zones is nearly equal, when transported to an intermediate climate; but that the former suffer principally from epidemics, an inference supported by some statements in Dr. Thomson's Essay.

In concluding this article, we must speak favorably of Dr. Thomson's Prize Thesis. It is a judicious summary of the facts best established in this department of medical science; and is no less creditable to himself than to the school whence it originates.

---

### ART. XII.

* Desgenettes, Histoire Médicale de l'Armée de l'Orient.

† Benoiston de Châteauneuf: Annales d'Hygiene, t. x., p. 2, 1833.

